# Comprehensive Immunogenetic Analysis of Long COVID Reveals HLA‐B*15 as an Independent Protective Factor

**DOI:** 10.1111/tan.71089

**Published:** 2026-11-13

**Authors:** José Luis Cano‐Perales, Pedro José Ortuño Pastor, Antonio Rosales‐Castillo, Mónica Bernal, Jose Ramón Vilchez, Pilar Jimenez, Ana Marín‐Sánchez, Antonio Rodriguez‐Nicolas, Asier Lizama‐Muñoz, Lucia Ballesta, Juan Sainz, Francisco Ruiz‐Cabello, Miguel Angel López‐Nevot, Juan Francisco Gutiérrez‐Bautista

**Affiliations:** ^1^ Servicio de Análisis Clínicos e Inmunología University Hospital Virgen de las Nieves Granada Spain; ^2^ Instituto de Investigación Biosanitaria de Granada (ibs.GRANADA) Granada Spain; ^3^ Servicio de Medicina Interna Hospital Universitario Virgen de las Nieves Granada Spain; ^4^ Laboratorio de Inmunología, Servicio de Hematología Hospital Regional Universitario Carlos Haya Malaga Spain; ^5^ Genomic Oncology Area, GENYO, Centre for Genomics and Oncological Research: Pfizer/University of Granada/Andalusian Regional Government, PTS Granada Spain; ^6^ Department of Biochemistry and Molecular Biology I, Faculty of Sciences University of Granada Granada Spain; ^7^ Departamento de Bioquímica, Biología Molecular e Inmunología III University of Granada Granada Spain

**Keywords:** HLA, HLA‐B*15, immunogenetics, long COVID, SARS‐CoV‐2

## Abstract

Long COVID is a frequent complication of SARS‐CoV‐2 infection, but the contribution of host immunogenetic factors to its development remains poorly understood. We investigated the association of HLA polymorphisms and other immunogenetic markers with Long COVID in a Spanish cohort. We performed a case–control study including 453 hospitalised patients with confirmed SARS‐CoV‐2 infection, of whom 168 developed Long COVID and 959 healthy controls. High‐resolution HLA typing was performed together with analyses of conserved extended haplotypes (CEHs), HLA‐B Bw4/Bw6 epitopes, HLA‐C C1/C2 ligand groups, MICA short tandem repeat polymorphisms and the CCR5 Δ32 variant. Logistic regression was used to identify independent predictors of Long COVID. After correction for multiple testing, no high‐resolution HLA allele remained significantly associated with Long COVID. However, the low‐resolution HLA‐B*15 serotype was significantly less frequent in patients with Long COVID and remained independently associated with a reduced risk of persistent symptoms after adjustment for age, sex and intensive care unit admission. The conserved extended haplotype CEH 44.2 was also less frequent among patients with Long COVID, although this association was not retained after multivariable adjustment. No significant associations were observed for HLA‐B Bw4/Bw6 epitopes, HLA‐C C1/C2 ligand groups, MICA polymorphisms or the CCR5 Δ32 variant. Our findings identify HLA‐B*15 as an independent protective immunogenetic factor against Long COVID and support a role for HLA‐mediated antiviral immunity in modulating susceptibility to persistent post‐COVID manifestations. These results warrant validation in larger, independent cohorts.

## 1 Introduction

Coronavirus disease 2019 (COVID‐19) is an infectious disease caused by severe acute respiratory syndrome coronavirus 2 (SARS‐CoV‐2), first identified in late 2019 and declared a global pandemic by the World Health Organization (WHO) in March 2020 [1]. The COVID‐19 pandemic was responsible for an estimated 15 million deaths worldwide during 2020–2021, including both direct COVID‐19‐related deaths and indirect mortality resulting from healthcare system disruption and reduced access to medical care [2, 3]. COVID‐19 typically presents as a respiratory illness with a broad spectrum of clinical severity, ranging from mild symptoms to severe disease, with older individuals and patients with underlying comorbidities being at greater risk of adverse outcomes [4]. Although most individuals recover completely without requiring hospitalisation, a substantial proportion experience persistent or newly developed symptoms beyond the acute phase of infection, a condition referred to as Long COVID, which may have a profound and long‐lasting impact on daily functioning [4, 5].

Long COVID encompasses a wide spectrum of clinical manifestations, including fatigue, cognitive impairment, dyspnea, musculoskeletal pain, autonomic dysfunction and a variety of neuropsychiatric symptoms [6]. The biological mechanisms underlying Long COVID remain incompletely understood and are likely multifactorial. Proposed mechanisms include viral persistence, immune dysregulation, chronic inflammation, endothelial dysfunction, reactivation of latent viruses, autoimmune processes and tissue damage resulting from the acute infection [7, 8]. The marked interindividual variability in susceptibility and clinical presentation suggests that host genetic factors may contribute to both the development and persistence of Long COVID [9, 10].

Among the genetic determinants of the immune response, the HLA system constitutes one of the most polymorphic regions of the human genome [11]. HLA molecules play a central role in antigen presentation and the initiation of adaptive immune responses by presenting pathogen‐derived peptides to T lymphocytes [11]. Variation in HLA alleles determines the repertoire of viral peptides that can be efficiently recognised by the immune system, thereby influencing susceptibility to infectious diseases, disease severity, vaccine responsiveness and the risk of developing autoimmune disorders [12, 13, 14]. Given its central role in antiviral immunity, the HLA system has been extensively investigated as a potential genetic determinant of susceptibility to SARS‐CoV‐2 infection and COVID‐19 severity [15].

Numerous studies have investigated the association between HLA alleles and susceptibility to SARS‐CoV‐2 infection or the severity of COVID‐19. Several Class I and Class II HLA alleles have been reported to confer either protection or increased susceptibility to severe disease, suggesting that interindividual differences in antigen presentation may influence the effectiveness of the antiviral immune response [16, 17, 18]. However, considerably less attention has been paid to the potential role of HLA variability in the development of Long COVID. Given the growing evidence implicating persistent immune activation, chronic inflammation and autoimmune‐like mechanisms in the pathogenesis of Long COVID, HLA polymorphisms may represent important determinants of individual susceptibility to this condition. Because HLA molecules determine antigen presentation to T lymphocytes, genetic variation within the HLA region may influence the quality and persistence of antiviral cellular immune responses following SARS‐CoV‐2 infection [19].

The identification of HLA alleles associated with Long COVID could contribute to a better understanding of the pathophysiology of this condition, facilitate risk stratification and help identify biologically distinct patient subgroups. Furthermore, such findings may provide valuable insights into the immunological mechanisms underlying symptom persistence and support the development of more targeted therapeutic strategies.

Beyond individual HLA alleles, conserved extended haplotypes (CEHs) may provide additional insight into the genetic basis of HLA‐disease associations. CEHs are long, highly conserved MHC haplotypes characterised by extensive linkage disequilibrium and encompass both HLA and non‐HLA immune‐related genes, including TNF, complement genes C4A/C4B and BTNL2 [20, 21, 22]. Thus, CEH analysis may help determine whether an association attributed to a classical HLA allele reflects the broader MHC haplotypic background or the effect of a closely linked functional variant.

In addition to classical HLA variation, other immune‐related polymorphisms may contribute to interindividual differences in the response to SARS‐CoV‐2. MICA is a stress‐inducible ligand of the activating NKG2D receptor expressed by NK and cytotoxic T cells, and variation in its exon 5 short tandem repeat (STR) has previously been associated with susceptibility to symptomatic SARS‐CoV‐2 infection [23]. CCR5, a chemokine receptor involved in immune‐cell trafficking and inflammatory responses, has also been implicated in COVID‐19, with previous studies investigating the potential effect of the functional CCR5 Δ32 deletion on susceptibility and disease severity [24, 25]. These observations provided the rationale for evaluating MICA STR and CCR5 Δ32 polymorphisms as additional candidate immunogenetic factors potentially associated with Long COVID.

Therefore, the aim of the present study was to investigate the association between immunogenetic factors and Long COVID by analysing HLA polymorphisms, conserved extended haplotypes, HLA homozygosity, HLA‐B Bw4/Bw6 epitopes, HLA‐C C1/C2 ligand groups, MICA polymorphisms and the CCR5 Δ32 variant in a cohort of individuals with confirmed SARS‐CoV‐2 infection.

## 2 Materials and Methods

### 2.1 Study Cohort

This retrospective observational cohort study included 453 consecutive adult patients hospitalised with COVID‐19 at Hospital Universitario Virgen de las Nieves (Granada, Spain) between April 2020 and January 2021 because of COVID‐19‐related pneumonia or acute respiratory failure. SARS‐CoV‐2 infection was confirmed by a positive RT‐PCR test. Baseline demographic and clinical characteristics of the study population are summarised in Table 1.

**TABLE 1:** Baseline demographic and clinical characteristics of the study cohort.

	Hospitalised COVID‐19 (*n* = 453)	Long COVID (*n* = 154)	No Long COVID (*n* = 229)	Healthy controls (*n* = 959)	*p* *
Age, years, mean ± SD	63.2 ± 15.2	58.1 ± 13.8	65 ± 15.5	45 ± 5	< 0.001
Female sex	220 (48.6)	76 (49.4)	136 (59.4)	489 (51)	n.s
ICU admission	126 (27.8)	47 (30.5)	35 (15.3)	N/A	< 0.001
Mechanical ventilation	101 (22.3)	42 (27.3)	20 (8.7)	N/A	< 0.001
In‐hospital mortality	70 (15.5)	N/A	N/A	N/A	N/A
Comorbidities					
Hypertension	196 (43.3)	62 (40.3)	94 (41)	N/A	n.s
Diabetes mellitus	101 (22.3)	30 (19.5)	49 (21.4)	N/A	n.s
Overweight or obesity	72 (15.9)	29 (18.8)	33 (14.4)	N/A	n.s
Chronic kidney disease	33 (7.3)	8 (5.2)	12 (5.2)	N/A	n.s
Chronic obstructive pulmonary disease	32 (7.1)	9 (5.8)	19 (8.3)	N/A	n.s
Asthma	26 (5.7)	7 (4.5)	13 (5.7)	N/A	n.s
Previous myocardial infarction	26 (5.7)	3 (1.9)	7 (3.1)	N/A	n.s
Heart failure	25 (5.5)	3 (1.9)	6 (2.6)	N/A	n.s
Cerebrovascular disease	22 (4.9)	5 (3.2)	7 (3.1)	N/A	n.s
Peripheral arterial disease	11 (2.4)	3 (1.9)	5 (2.2)	N/A	n.s

*Note:* Data are presented as mean ± standard deviation (SD) for continuous variables and as number (percentage) for categorical variables. Long COVID status was assessed among the 383 patients who survived the acute infection (154 with Long COVID and 229 without Long COVID); the overall hospitalised COVID‐19 cohort (*n* = 453) also included 70 patients who died during hospitalisation. Healthy controls were unrelated volunteer blood donors from the same geographical area, with no known comorbidities at the time of donation, and were included as a population reference group for the immunogenetic analyses rather than as a clinical comparator.

Abbreviations: ICU, intensive care unit; N/A, not applicable or data not available; n.s., not significant; SD, standard deviation.

* *p* values correspond to comparisons between patients with and without Long COVID.

A total of 959 unrelated healthy volunteer blood donors from the Granada region were included as controls. All controls were of self‐reported European ancestry, had no known comorbidities at the time of donation, and were free of SARS‐CoV‐2 infection at the time of sample collection. These individuals had previously been recruited for immunogenetic studies performed by our group and were included in the present study as a population reference group for the immunogenetic analyses. HLA typing was performed using the same methodology as that applied to the patient cohort. The control cohort had a mean age of 45 ± 5 years, with females representing 51% of the study population. HLA‐DQA1 and HLA‐DPB1 typing was not available for the healthy control group; therefore, analyses involving these loci were restricted to the patient cohort.

The study was approved by the Biomedical Research Ethics Committee of Andalusia (approval code 0766‐N‐20), and written informed consent was obtained from all participants prior to inclusion.

### 2.2 Long COVID

Long COVID was defined as cases with microbiological confirmation of SARS‐CoV‐2 infection and the presence of persistent symptoms for at least 3 months affecting one or more organ systems, in accordance with the definition of the National Academies of Sciences, Engineering, and Medicine (NASEM) [5]. Cases with an alternative explanation for the reported symptoms, incomplete medical records or absence of microbiological confirmation of prior SARS‐CoV‐2 infection were excluded.

Because Long COVID can only be assessed in patients who survive the acute phase of SARS‐CoV‐2 infection and have adequate clinical follow‐up, the study population for the HLA association analyses consisted of all patients who survived the acute episode and for whom Long COVID status could be determined.

Long COVID was identified in 154 patients, corresponding to 34% of the study population. After excluding patients who died, the proportion of survivors who developed long COVID increased to 40.2%. The baseline demographic and clinical characteristics of patients with long COVID are presented in Table 2.

**TABLE 2:** Baseline characteristics and persistent clinical manifestations of patients with long COVID.

Variable	Value
Age, years	58 (38–78)
Female sex, *n* (%)	76 (49.4)
ICU admission, *n* (%)	48 (31.2)
Mechanical ventilation, *n* (%)	17 (11)
Clinical manifestation	*n* (%)
Respiratory symptoms	130 (84.4)
Cough	9 (5.8)
Dyspnea	66 (42.9)
Chest pain	6 (3.9)
More than one respiratory symptom	49 (31.8)
Gastrointestinal symptoms	11 (7.1)
Diarrhoea	7 (4.5)
Abdominal pain	2 (1.3)
Constipation	0 (0.0)
More than one gastrointestinal symptom	2 (1.3)
Cardiovascular symptoms	22 (14.3)
Tachycardia/palpitations	15 (9.7)
Orthostatic intolerance	0 (0.0)
Hypertension	5 (3.2)
More than one cardiovascular symptom	2 (1.3)
Neurological symptoms	62 (40.3)
Brain fog	4 (2.6)
Memory impairment	15 (9.7)
Headache	12 (7.8)
More than one neurological symptom	30 (19.5)
Other neurological symptoms	1 (0.6)
Dizziness/Syncope	16 (10.4)
Fatigue/Asthenia	109 (70.8)
Post‐exertional malaise	45 (29.2)
Musculoskeletal symptoms	54 (35.1)
Myalgia	20 (13.0)
Arthralgia	13 (8.4)
More than one musculoskeletal symptoms	21 (13.6)
Taste and/or smell disorders	16 (10.4)
Dysgeusia	3 (1.9)
Hyposmia	5 (3.2)
Both dysgeusia and hyposmia	8 (5.2)
Anxiety/Depression	35 (22.7)
Sleep disorders (insomnia)	23 (14.9)

*Note:* Values are presented as median (range) or *n* (%). Percentages were calculated using the total number of patients with long COVID (*n* = 154). Patients classified as ‘more than one symptom’ reported two or more manifestations within the corresponding symptom category. The complete symptom checklist used for data collection is provided in Supplementary Table S1.

Persistent symptoms were systematically assessed during follow‐up using a predefined symptom checklist and subsequently grouped by organ system for presentation in Table 2. The complete symptom checklist is provided in Supplementary Table S1.

### 2.3 SARS‐CoV‐2 Diagnosis

SARS‐CoV‐2 infection was confirmed in all participants by reverse transcription polymerase chain reaction (RT‐PCR) performed on respiratory specimens (nasal, nasopharyngeal or oropharyngeal swabs). Molecular testing was carried out using the cobas SARS‐CoV‐2 assay on the cobas 6800 System (Roche Molecular Systems, Pleasanton, CA, USA).

### 2.4 DNA Extraction and HLA Genotyping

Genomic DNA was extracted from peripheral blood. DNA was isolated using the QIAamp DNA Blood Mini Kit (Qiagen, Hilden, Germany) following the manufacturer's recommendations. High‐resolution genotyping of HLA Class I (HLA‐A, HLA‐B and HLA‐C) and Class II (HLA‐DRB1, HLA‐DQB1 and HLA‐DPB1) loci was performed using the LABType sequence‐specific oligonucleotide (SSO) typing assay (One Lambda, Canoga Park, CA, USA). After PCR amplification with locus‐specific primers, amplified products were hybridised with allele‐specific oligonucleotide probes coupled to fluorescent microspheres. Fluorescence signals were acquired on a LABScan 100 platform (Luminex xMAP, Austin, TX, USA), and HLA genotypes were assigned using HLA Fusion software (Palex). Alleles were assigned according to the current IMGT/HLA nomenclature.

Conserved extended haplotypes (CEH) were assigned according to the definitions previously reported by MT Dorak et al. [20].

The presence of Bw4 and Bw6 epitopes was determined according to the epitope motifs encoded by HLA‐A and HLA‐B alleles, as previously described [26]. Individuals were classified according to their Bw4/Bw6 phenotype as Bw4/Bw4 homozygotes, Bw6/Bw6 homozygotes or Bw4/Bw6 heterozygotes.

HLA‐C alleles were assigned to the C1 or C2 ligand groups according to their KIR‐binding specificity [27]. Based on their HLA‐C genotype, individuals were classified as C1/C1 homozygotes, C2/C2 homozygotes or C1/C2 heterozygotes.

### 2.5 MICA Genotyping

The study of the STR polymorphism of exon 5 of MICA (major histocompatibility complex Class I chain‐related A) was performed by polymerase chain reaction (PCR) following the method of Ota et al. [28].

### 2.6 CCR5 Δ32 Genotyping

CCR5‐Δ32 genotyping was performed by PCR amplification followed by fragment analysis, distinguishing the 174‐bp wild‐type allele from the 142‐bp deletion allele by capillary electrophoresis, following the method of Veloso et al. [29].

### 2.7 Statistical Analysis

Allelic, genotypic and haplotypic association analyses were conducted using the BIGDAWG package implemented in the R statistical environment (R Foundation for Statistical Computing, Vienna, Austria). Hardy–Weinberg equilibrium (HWE) was assessed separately for HLA‐A, HLA‐B, HLA‐C, HLA‐DRB1 and HLA‐DQB1 in the healthy control group using PyPop (version 0.7.0). Differences in HLA allele frequencies between cases and controls were evaluated using Pearson's *χ* ^2^ test. To ensure the validity of the analyses, alleles with expected cell counts below five were pooled into a single category before statistical testing.

Permutation‐based empirical *p* values were calculated using BIGDAWG with 100,000 permutations. Group labels were randomly permuted while preserving individual HLA genotypes, and the association analysis was repeated for each permuted dataset to generate the null distribution. Empirical *p* values were estimated from the proportion of permuted results at least as extreme as the observed association.

Variables associated with Long COVID in univariate analyses were considered for multivariable analysis. Age, sex and ICU admission were included as covariates in the adjusted models. Mechanical ventilation was not included simultaneously with ICU admission because of the strong overlap between both measures of acute disease severity. Adjusted odds ratios (ORs) and 95% confidence intervals (95% CIs) were calculated. Multivariable logistic regression analyses were performed using IBM SPSS Statistics for Windows, version 26.0 (IBM Corp., Armonk, NY, USA).

Continuous variables were summarised as median (range), whereas categorical variables were expressed as counts and percentages.

Association analyses were performed by comparing (i) patients with Long COVID versus patients without Long COVID and (ii) patients with Long COVID versus healthy controls.

For multiple‐testing correction, the numbers of alleles evaluated at high resolution were 17 for HLA‐A, 44 for HLA‐B, 18 for HLA‐C, 37 for HLA‐DRB1, 14 for HLA‐DQB1, 10 for HLA‐DQA1 and 14 for HLA‐DPB1. At low resolution, the corresponding numbers were 14, 21, 13, 13, 5, 5 and 11, respectively. To account for multiple comparisons, *p* values were adjusted using the Bonferroni correction, and corrected *p* values < 0.05 were considered statistically significant throughout the study.

## 3 Results

Among the 383 COVID‐19 survivors included in the analysis, 154 (40.2%) developed Long COVID. Patients with Long COVID were significantly younger than those without persistent symptoms (median age, 58 vs. 66 years; *p* = 1.2 × 10^−5^). Patients who died during hospitalisation were significantly older than those who survived (68.76 ± 14.18 vs. 62.19 ± 15.20 years, respectively; *p* = 0.001). Among survivors, when age was analysed categorically, the proportion of patients developing Long COVID progressively decreased across age groups: 53.8% in patients aged < 50 years, 48.8% in those aged 50–59 years, 39.1% in those aged 60–69 years and 27.3% in those aged ≥ 70 years (*p* < 0.001). This association remained significant after adjustment for sex and ICU admission (overall *p* = 0.001). Using patients aged < 50 years as the reference group, the adjusted ORs were 0.813 (95% CI: 0.432–1.532; *p* = 0.522) for 50–59 years, 0.500 (95% CI: 0.264–0.946; *p* = 0.033) for 60–69 years and 0.318 (95% CI: 0.176–0.577; *p* < 0.001) for patients aged ≥ 70 years.

ICU admission (31.2% vs. 15.7%; *p* = 3.4 × 10^−4^) and the need for mechanical ventilation (11.0% vs. 4.4%; *p* = 0.002) were also significantly more frequent among patients with Long COVID. Although the proportion of women was higher in the Long COVID group (49.4% vs. 40.4%), the difference did not reach statistical significance.

A multivariable logistic regression analysis including age, sex and ICU admission demonstrated that younger age (adjusted OR = 0.969 per year, *p* < 0.001), female sex (male sex: adjusted OR = 0.560, *p* = 0.010), and ICU admission (adjusted OR = 2.754, *p* < 0.001) were independently associated with Long COVID (Table 3). Additional models showed that the association with sex emerged after adjustment for either age (OR = 0.652, *p* = 0.049) or ICU admission (OR = 0.591, *p* = 0.017), suggesting that both variables contributed to unmasking the association observed in the fully adjusted model.

**TABLE 3:** Multivariable logistic regression analysis of factors associated with Long COVID.

Variable	Adjusted OR	95% CI	*p*
Age (per year)	0.969	0.955–0.983	< 0.001
Male sex	0.560	0.360–0.872	0.010
ICU admission	2.754	1.636–4.636	< 0.001

Abbreviation: ICU, intensive care unit.

No significant deviations from Hardy–Weinberg equilibrium were observed for any of the HLA loci evaluated in the healthy control group (HLA‐A, *p* = 0.9113; HLA‐B, *p* = 0.9179; HLA‐C, *p* = 0.9728; HLA‐DRB1, *p* = 0.1116 and HLA‐DQB1, *p* = 0.8259).

High‐resolution HLA allele frequencies were compared between patients with Long COVID, patients without Long COVID and healthy controls (Table 4). No deviations from Hardy–Weinberg equilibrium were observed. Several HLA alleles showed lower frequencies among patients with Long COVID compared with either healthy controls or patients without Long COVID, including HLA‐A*32:01, HLA‐B*15:01, HLA‐B*44:03 and HLA‐DRB1*04:01. Conversely, HLA‐B*14:02 and HLA‐DQA1*03:02 were more frequent in patients with Long COVID than in healthy controls and patients without Long COVID, respectively (Table 4). However, none of these associations remained statistically significant after Bonferroni correction.

**TABLE 4:** Comparison of high‐resolution HLA allele frequencies between patients with and without Long COVID and healthy controls.

HLA allele	Long COVID (*n* = 154)	No Long COVID (*n* = 229)	Controls (*n* = 959)	P1 (Pc)	Empirical P1	P2 (Pc)	Empirical P2
HLA‐A*01:01	0.127	0.098	0.100	n.s	n.s	n.s	n.s
HLA‐A*24:02	0.084	0.100	0.106	n.s	n.s	n.s	n.s
HLA‐A*32:01	0.019	0.055	0.033	0.016 (n.s)	0.020	n.s	n.s
HLA‐A*66:01	0.019	0.011	0.004	n.s	n.s	n.s	n.s
HLA‐B*14:02	0.068	0.041	0.036	n.s	n.s	0.012 (n.s)	0.014
HLA‐B*15:01	0.001	0.031	0.031	n.s	n.s	0.046 (n.s)	0.043
HLA‐B*18:01	0.084	0.083	0.103	n.s	n.s	n.s	n.s
HLA‐B*44:03	0.084	0.138	0.097	0.024 (n.s)	0.030	n.s	n.s
HLA‐B*50:01	0.039	0.017	0.028	n.s	n.s	n.s	n.s
HLA‐C*06:02	0.104	0.064	0.080	n.s	n.s	n.s	n.s
HLA‐C*16:01	0.075	0.109	0.075	n.s	n.s	n.s	n.s
HLA‐DRB1*01:02	0.036	0.022	0.032	n.s	n.s	n.s	n.s
HLA‐DRB1*03:01	0.117	0.157	0.116	n.s	n.s	n.s	n.s
HLA‐DRB1*04:01	0.006	0.022	0.028	n.s	n.s	0.022 (n.s)	0.042
HLA‐DRB1*07:01	0.149	0.181	0.164	n.s	n.s	n.s	n.s
HLA‐DRB1*11:01	0.039	0.052	0.068	n.s	n.s	n.s	n.s
HLA‐DRB1*15:01	0.075	0.076	0.095	n.s	n.s	n.s	n.s
HLA‐DQB1*02:01	0.117	0.155	0.117	n.s	n.s	n.s	n.s
HLA‐DQB1*02:02	0.123	0.155	0.138	n.s	n.s	n.s	n.s
HLA‐DQB1*03:01	0.192	0.159	0.179	n.s	n.s	n.s	n.s
HLA‐DQB1*06:02	0.068	0.066	0.091	n.s	n.s	n.s	n.s
HLA‐DQA1*03:02	0.026	0.004	—	0.018 (n.s)	0.008	—	—
HLA‐DQA1*05:01	0.136	0.177	—	n.s	n.s	—	—
HLA‐DQA1*05:05	0.136	0.105	—	n.s	n.s	—	—
HLA‐DPB1*04:01	0.260	0.314	—	n.s	n.s	—	—
HLA‐DPB1*12:01	0.143	0.122	—	n.s	n.s	—	—

*Note:* P1, Long COVID vs. No Long COVID; P2, Long COVID vs. healthy controls; Pc, Bonferroni‐corrected *p*‐value. Empirical *p* values were calculated using BIGDAWG with 100,000 permutations. Selected alleles showing the most notable frequency differences are displayed; all alleles were included in the statistical analyses.

Abbreviation: n.s., not significant.

To reduce the number of alleles analysed and increase statistical power, HLA allele frequencies were subsequently evaluated at low‐resolution level (Table 5). Although several alleles showed differences in frequency between groups, only HLA‐B*15 remained significantly associated with Long COVID after Bonferroni correction. HLA‐B*15 was less frequent among patients with Long COVID than among healthy controls (Pc = 0.042; OR = 0.24, 95% CI: 0.09–0.67), indicating a potential protective association.

**TABLE 5:** Comparison of low‐resolution HLA allele frequencies in Long COVID, non‐Long COVID and healthy controls.

HLA allele	Long COVID (*n* = 154)	No Long COVID (*n* = 229)	Controls (*n* = 959)	P1 (Pc)	Empirical P1	P2 (Pc)	Empirical P2
HLA‐A*01	0.127	0.099	0.102	n.s	n.s	n.s	n.s
HLA‐A*02	0.269	0.242	0.262	n.s	n.s	n.s	n.s
HLA‐A*32	0.019	0.055	0.034	0.016 (n.s)	0.020	n.s	n.s
HLA‐B*15	0.013	0.044	0.052	0.019 (n.s)	0.021	0.002 (0.042)	0.002
HLA‐B*44	0.114	0.188	0.154	0.006 (n.s)	0.012	n.s	n.s
HLA‐B*55	0.023	0.015	0.070	n.s	n.s	0.014 (n.s)	0.009
HLA‐C*03	0.032	0.066	0.045	0.044 (n.s)	0.047	n.s	n.s
HLA‐C*06	0.104	0.066	0.081	n.s	n.s	n.s	n.s
HLA‐C*16	0.088	0.124	0.090	n.s	n.s	n.s	n.s
HLA‐DRB1*01	0.114	0.083	0.123	n.s	n.s	n.s	n.s
HLA‐DRB1*03	0.118	0.157	0.116	n.s	n.s	n.s	n.s
HLA‐DRB1*04	0.114	0.109	0.131	n.s	n.s	n.s	n.s
HLA‐DRB1*07	0.150	0.181	0.164	n.s	n.s	n.s	n.s
HLA‐DQB1*02	0.240	0.310	0.256	n.s	n.s	n.s	n.s
HLA‐DQB1*03	0.318	0.286	0.311	n.s	n.s	n.s	n.s
HLA‐DQB1*06	0.192	0.188	0.212	n.s	n.s	n.s	n.s
HLA‐DQA1*01	0.391	0.371	—	n.s	n.s	n.s	n.s
HLA‐DQA1*02	0.145	0.166	—	n.s	n.s	n.s	n.s
HLA‐DPB1*02	0.214	0.174	—	n.s	n.s	n.s	n.s
HLA‐DPB1*04	0.433	0.453	—	n.s	n.s	n.s	n.s
HLA‐DPB1*11	0.034	0.052	—	n.s	n.s	n.s	n.s

*Note:* P1, Long COVID vs No Long COVID; P2, Long COVID vs healthy controls; Pc, Bonferroni‐corrected *p*‐value. Empirical *p* values were calculated using BIGDAWG with 100,000 permutations. Selected alleles showing the most notable frequency differences are displayed; all alleles were included in the statistical analyses.

Abbreviation: n.s., not significant.

To determine whether this association was independent of demographic and clinical factors, HLA‐B*15 carriage was included in the multivariable logistic regression model. The protective effect of HLA‐B*15 remained statistically significant after adjustment for age, sex and ICU admission (adjusted OR = 0.212, 95% CI: 0.059–0.757; *p* = 0.017), indicating that this association was independent of the main clinical predictors of Long COVID (Table 6).

**TABLE 6:** Multivariable logistic regression of genetic marker associated with Long COVID.

	aOR	95% CI	*p*
HLA‐A*32:01	0.289	0.108–0.777	0.014
HLA‐B*44:03	0.556	0.321–0.964	0.037
HLA‐DQA1*03:02	6.978	1.406–34.626	0.017
HLA‐A*32	0.289	0.108–0.777	0.014
HLA‐B*15	0.277	0.089–0.865	0.027
HLA‐B*44	0.558	0.337–0.926	0.024
HLA‐C*03	0.364	0.159–0.834	0.017
CEH 44.2	1.246	0.603–2.576	0.552

*Note:* Each genetic marker was evaluated in a separate multivariable logistic regression model adjusted for age, sex and ICU admission.

Abbreviations: aOR, adjusted odds ratio; CEH, conserved extended haplotype; CI, confidence interval.

In separate multivariable logistic regression models adjusted for age, sex and ICU admission, the HLA associations identified in the Long COVID versus No Long COVID comparison remained nominally significant, including HLA‐A*32:01, HLA‐B*44:03 and HLA‐DQA1*03:02 at high resolution, and HLA‐A*32, HLA‐B*15, HLA‐B*44 and HLA‐C*03 at low resolution (Table 6). Notably, HLA‐B*15 remained independently associated with a reduced likelihood of Long COVID (aOR = 0.277, 95% CI: 0.089–0.865; *p* = 0.027). However, these adjusted associations should be interpreted as nominal, since HLA‐B*15 was the only allele‐level association that remained significant after correction for multiple comparisons in the primary allele‐frequency analyses.

Finally, the distribution of conserved extended HLA haplotypes was analysed (Table 7). The frequency of the conserved extended haplotype CEH 44.2 (HLA‐A*29:02~C*16:01~B*44:03~DRB1*07:01~DQA1*02:01~DQB1*02:02) was significantly lower in patients with Long COVID than in patients without Long COVID (OR = 0.24, 95% CI: 0.08–0.71), and this association remained statistically significant after Bonferroni correction. In addition, the CEH 7.1 (HLA‐A*03:01~C*07:02~B*07:02~DRB1*15:01~DQA1*01:02~DQB1*06:02) and CEH 8.1 (HLA‐A*01:01~C*07:01~B*08:01~DRB1*03:01~DQA1*05:01~DQB1*02:01) haplotypes were not detected among patients with Long COVID, although these differences did not remain significant after correction for multiple comparisons. To determine whether the association between the CEH 44.2 haplotype and Long COVID was independent of demographic and clinical factors, CEH 44.2 carriage was included in the multivariable logistic regression model. After adjustment for age, sex and ICU admission, the association was no longer statistically significant (adjusted OR = 0.823, 95% CI: 0.418–1.621; *p* = 0.573).

**TABLE 7:** Frequencies of conserved extended HLA haplotypes in patients with and without Long COVID and healthy controls.

Haplotype	Long COVID (*n* = 154)	No Long COVID (*n* = 229)	Controls (*n* = 959)	P1 (Pc)	P2 (Pc)
7.1	0	0.026	0.030	n.s	0.034 (n.s)
8.1	0	0.035	0.036	0.024 (n.s)	0.013 (n.s)
44.2	0.026	0.100	0.055	0.004 (0.024)	n.s

*Note:* P1: Long COVID vs. No long COVID; P2: Long COVID vs. Control. Pc: P corrected by Bonferroni; Haplotype 7.1: HLA‐A*03:01~C*07:02~B*07:02~DRB1*15:01~DQA1*01:02~DQB1*06:02; Haplotype 8.1: HLA‐A*01:01~C*07:01~B*08:01~DRB1*03:01~DQA1*05:01~DQB1*02:01; Haploype 44.2: HLA‐A*29:02~C*16:01~B*44:03~DRB1*07:01~DQA1*02:01~DQB1*02:02.

Abbreviation: n.s., not significant.

Finally, no significant differences were observed between patients with Long COVID, patients without Long COVID and controls in the distribution of HLA‐Bw4/Bw6 epitopes, HLA‐C C1/C2 ligand groups, CCR5 Δ32 polymorphism or MICA STR variants. Detailed results of these analyses are provided in Supplementary Tables [Link], [Link], [Link].

## 4 Discussion

In the present study, we investigated the association between immunogenetic factors and the development of Long COVID in a cohort of patients with confirmed SARS‐CoV‐2 infection. Our findings indicate that, while the overall distribution of HLA alleles showed limited differences between patients with and without Long COVID after correction for multiple comparisons, specific immunogenetic features, particularly the conserved extended haplotype CEH 44.2 and HLA‐B*15 serotype, were associated with a reduced risk of developing persistent post‐COVID symptoms. Furthermore, no significant associations were observed for Bw4/Bw6 epitopes, C1/C2 groups, MICA polymorphisms or the CCR5 Δ32 variant.

Before discussing the immunogenetic findings, it is important to consider the clinical characteristics of the study population. Our findings are consistent with previous evidence demonstrating that the severity of acute COVID‐19 is associated with an increased risk of Long COVID. Hospitalisation, severe disease and the need for intensive care or mechanical ventilation have all been linked to persistent post‐COVID symptoms, in agreement with our observation that ICU admission and mechanical ventilation were significantly more frequent among patients who developed Long COVID [30, 31]. Several studies have identified female sex as a risk factor for the development of Long COVID [32, 33, 34, 35]. Although no significant differences in sex distribution were observed in the univariate analysis, female sex was independently associated with Long COVID after adjustment for age and disease severity in the multivariable logistic regression model. In contrast, patients with Long COVID in our cohort were significantly younger than those without persistent symptoms, and this association remained significant after adjustment for sex and ICU admission. Although this finding differs from that reported in several previous studies, the role of age as an independent risk factor for Long COVID remains controversial [30, 33]. The inverse association between age and Long COVID should nevertheless be interpreted in light of a potential survivor selection effect, since Long COVID could only be assessed among patients who survived the acute infection. Patients who died during hospitalisation were significantly older than survivors, suggesting that the restriction of the Long COVID analysis to survivors may have contributed to the observed association with younger age. However, when age was analysed categorically among survivors, the prevalence of Long COVID progressively decreased across increasing age groups, and the association remained significant after adjustment for sex and ICU admission. Thus, although survivor selection may have influenced the magnitude of the association, the age‐stratified analysis supports an inverse association between age and Long COVID within the survivor cohort. Overall, the demographic and clinical characteristics of our cohort are largely consistent with those described in previous studies, providing additional support for the appropriateness of the patient selection criteria used to define Long COVID in this study.

The main immunogenetic finding of our study was the protective association between HLA‐B*15 and the development of Long COVID. This association remained significant after adjustment for age, sex and ICU admission, suggesting that HLA‐B*15 may represent an independent protective factor against persistent post‐COVID symptoms. Although the role of HLA‐B*15 has not been extensively investigated in Long COVID, previous studies have consistently associated this allele, particularly HLA‐B*15:01, with asymptomatic SARS‐CoV‐2 infection, milder clinical disease, and improved survival [16, 36, 37]. These observations suggest that HLA‐B*15 may contribute to a more efficient antiviral immune response during the acute phase of infection [16]. The proposed mechanism underlying this protective effect is related to the ability of HLA‐B*15, particularly HLA‐B*15:01, to efficiently present highly conserved SARS‐CoV‐2 peptides to CD8^+^ T lymphocytes. Individuals carrying this allele have been shown to possess pre‐existing, polyfunctional memory CD8^+^ T cells that are cross‐reactive with homologous peptides from seasonal human coronaviruses, potentially enabling a faster and more effective antiviral immune response during the early stages of SARS‐CoV‐2 infection [16]. Recent mechanistic evidence further supports this hypothesis. Rowntree et al. demonstrated that HLA‐B*15:01‐positive individuals with mild COVID‐19 preferentially expanded public TRAV9‐2/TRBV7‐2 CD8^+^ T‐cell receptor clonotypes specific for the conserved Spike S919 epitope, whereas HLA‐B*15:01‐positive patients with severe disease lacked these highly expanded clonotypes and instead exhibited an alternative T‐cell receptor repertoire [38]. These findings suggest that the protective effect of HLA‐B*15 depends not only on antigen presentation itself but also on the quality and clonotypic composition of the antiviral CD8^+^ T‐cell response, providing a plausible biological explanation for the variability in clinical outcomes among HLA‐B*15 carriers [38]. It is conceivable that a more efficient early cellular immune response may promote faster viral clearance, thereby limiting prolonged antigen exposure and the sustained immune activation that have been proposed as key mechanisms in the pathogenesis of Long COVID [7, 39]. Although our study cannot establish the underlying mechanism, the independent protective association observed for HLA‐B*15 is consistent with the hypothesis that host genetic factors influencing the quality of the early antiviral T‐cell response may also modulate the risk of developing persistent post‐COVID symptoms.

In the multivariable analyses, several HLA markers remained nominally associated with Long COVID after adjustment for age, sex and ICU admission, suggesting that these associations were not solely explained by differences in demographic characteristics or acute disease severity. However, these findings should be interpreted cautiously, as most of these associations did not withstand correction for multiple comparisons in the primary allele‐frequency analyses. Notably, HLA‐B*15 showed the most robust evidence of association, as it remained independently associated with a reduced likelihood of Long COVID after clinical adjustment and was the only allele‐level marker to retain statistical significance after correction for multiple comparisons. Thus, HLA‐B*15 represents the principal immunogenetic signal identified in our study, whereas the remaining HLA associations should be considered exploratory and require validation in independent cohorts.

Only a limited number of studies have investigated the role of HLA polymorphisms in Long COVID, and the reported associations have been highly heterogeneous. Most candidate‐gene studies published to date have included relatively small cohorts, generally smaller than the population analysed in the present study, which may have contributed to the lack of reproducibility across studies. Different HLA alleles have been associated with susceptibility or protection, including HLA‐A*11, HLA‐C*07, HLA‐A*03 and HLA‐G polymorphisms [40, 41, 42], whereas more recent genome‐wide analyses have identified significant signals within the HLA‐DQA1/HLA‐DQB1 region [43]. However, no single HLA allele has been consistently replicated across independent populations, suggesting that differences in ancestry, sample size, study design and Long COVID definition may substantially influence the observed associations. In this context, our findings provide further evidence supporting the involvement of the HLA region in Long COVID susceptibility while identifying HLA‐B*15 as a potential protective factor that warrants validation in larger independent cohorts.

In our study, no HLA allele reached statistical significance after correction for multiple comparisons at high‐resolution typing. However, HLA‐B*15:01 showed a trend towards significance, consistent with the protective association observed for the corresponding low‐resolution serotype, HLA‐B*15. This finding suggests that the lack of significance at high resolution may be related to the limited sample size, as the subdivision of HLA‐B*15 into multiple alleles reduces the number of carriers in each subgroup. Larger cohorts with high‐resolution HLA typing will be required to confirm whether HLA‐B*15:01 or other HLA‐B*15 alleles are responsible for the protective effect observed in our study.

HLA‐DRB1*04:01 also showed a nominal association in our high‐resolution analysis, although this did not remain significant after correction for multiple comparisons. This finding is of interest given the report by Augusto et al. that HLA‐DRB1*04:01 enhanced the association between HLA‐B*15:01 and asymptomatic SARS‐CoV‐2 infection [16]. However, only one individual in our cohort carried both HLA‐B*15:01 and HLA‐DRB1*04:01, precluding a meaningful assessment of their combined effect in Long COVID.

In addition to HLA‐B*15, the conserved extended haplotype CEH 44.2 was significantly less frequent among patients with Long COVID than among patients without persistent symptoms. This association remained significant after correction for multiple comparisons, suggesting a potential protective role of this haplotype. However, when demographic and clinical variables were included in the multivariable logistic regression model, the association was no longer statistically significant. These findings indicate that the protective effect observed in the univariate analysis may be influenced, at least in part, by differences in the clinical characteristics of the study population. Although CEH 44.2 has not previously been investigated in the context of Long COVID, our group has recently reported that its Class II component, comprising HLA‐DRB1*07:01~HLA‐DQA1*02:01~HLA‐DQB1*02:02, was associated with the magnitude of the humoral immune response following mRNA‐1273 vaccination [44]. This observation supports the hypothesis that genetic variation within this conserved haplotype, particularly within the HLA Class II region, may influence adaptive immune responses to SARS‐CoV‐2.

Finally, we investigated additional immunogenetic markers potentially involved in the host response to SARS‐CoV‐2, including HLA‐B Bw4/Bw6 epitopes, HLA‐C C1/C2 ligand groups, MICA polymorphisms, and the CCR5 Δ32 variant. None of these markers showed significant differences between patients with and without Long COVID, suggesting that their contribution to the development of persistent post‐COVID symptoms is limited in our study population. Although these molecules have been implicated in antiviral immunity through the regulation of natural killer cell activity, antigen presentation or leukocyte trafficking, our results do not support a major role for these immunogenetic factors in determining Long COVID susceptibility.

Taken together, our findings suggest that classical HLA polymorphism, particularly HLA‐B*15, may have a greater influence on Long COVID susceptibility than other immunogenetic markers evaluated in the present study.

This study has several limitations that should be considered when interpreting the results. First, although our cohort is larger than those included in most previous candidate‐gene studies of HLA and Long COVID, the sample size remains relatively modest for genetic association analyses, particularly after stratification into individual high‐resolution HLA alleles. This limited statistical power may have reduced our ability to detect associations with low‐frequency alleles and could explain why some findings, such as the association with HLA‐B*15:01, did not reach statistical significance despite showing the same trend as the corresponding low‐resolution serotype. Second, our findings were obtained in a single‐center cohort of predominantly European ancestry and therefore require external validation in independent populations with different genetic backgrounds. Finally, although we adjusted the main analyses for relevant clinical variables, including age, sex and disease severity, additional environmental and host‐related factors may also contribute to the development of Long COVID.

Despite these limitations, our study represents one of the most comprehensive immunogenetic analyses of Long COVID to date, integrating high‐ and low‐resolution HLA typing, conserved extended haplotypes, HLA homozygosity, HLA‐B Bw4/Bw6 epitopes, HLA‐C C1/C2 ligand groups, MICA polymorphisms and the CCR5 Δ32 variant within the same cohort. Our findings support a potential protective role of HLA‐B*15 against Long COVID and reinforce the relevance of the HLA region in modulating the host response to SARS‐CoV‐2. Future multicenter studies including larger cohorts, high‐resolution HLA typing and functional characterisation of antigen‐specific T‐cell responses will be essential to validate these associations and to better understand the immunological mechanisms underlying Long COVID susceptibility.

## Author Contributions

Conceptualisation: F.R.‐C., M.A.L.‐N., J.F.G.‐B. Methodology: J.R.V., A.R.‐C., M.A.L.‐N., J.F.G.‐B. Investigation: J.L.C.‐P., P.J.O.P., M.B., J.R.V., P.J., A.M.‐S., A.R.‐N., A.L.‐M., L.B., J.S. Patient recruitment and clinical data collection: P.J.O.P., A.R.‐C., J.L.C.‐P., J.R.V., J.F.G.‐B., A.M.‐S., L.B. Laboratory analyses and HLA genotyping: J.L.C.‐P., A.L.‐M., J.R.V., A.R.‐N. Formal analysis: J.F.G.‐B. Data curation: J.L.C.‐P., P.J.O.P., A.R.‐C., J.F.G.‐B. Writing – original draft: J.L.C.‐P., P.J.O.P., A.R.‐C., J.F.G.‐B. Writing – review and editing: All authors. Supervision: F.R.‐C., M.A.L.‐N., A.R.‐C., J.F.G.‐B.

## Funding

This work was supported by Instituto de Salud Carlos III (JR25/00046).

## Ethics Statement

This study was conducted in accordance with the principles of the Declaration of Helsinki and was approved by the Biomedical Research Ethics Committee of Andalusia (approval code 0766‐N‐20), and written informed consent was obtained from all participants prior to inclusion.

## Conflicts of Interest

The authors declare no conflicts of interest.

## Supporting information


**Supplementary Table 1:** Symptom checklist used for the clinical assessment of Long COVID.

**Supplementary Table 2:** Distribution of Bw4/Bw6 epitopes and HLA‐C C1/C2 groups in patients with and without Long COVID and healthy controls.

**Supplementary Table 3:** Distribution of MICA STR alleles in patients with and without Long COVID and healthy controls.

**Supplementary Table 4:** Distribution of CCR5 Δ32 genotypes in patients with and without Long COVID.

## Data Availability

The data that support the findings of this study are available on request from the corresponding author. The data are not publicly available due to privacy or ethical restrictions.
