# Supplementary material for: Comprehensive Immunogenetic Analysis of Long COVID Reveals HLA‐B*15 as an Independent Protective Factor
Source: HLA. 2026 Nov 13;108(5):e71089. doi: 10.1111/tan.71089 (PMC13762371; doi:10.1111/tan.71089)
Supplement: Supplementary file 1 [file TAN-108-e71089-s001.docx]

**Supplementary Table 3.** Distribution of MICA STR alleles in patients with and without Long COVID and healthy controls.

| MICA STR allele | Long COVID (n=154) | No Long COVID (n=229) | Controls (n=959) | P1 (Pc) | P2 (Pc) |
| --- | --- | --- | --- | --- | --- |
| MICA*4 | 0.139 | 0.136 | 0.151 | n.s | n.s |
| MICA*5 | 0.106 | 0.116 | 0.120 | n.s | n.s |
| MICA*5.1 | 0.219 | 0.209 | 0.251 | n.s | n.s |
| MICA*6 | 0.348 | 0.338 | 0.344 | n.s | n.s |
| MICA*9 | 0.175 | 0.196 | 0.134 | n.s | n.s |

MICA STR: short tandem repeat polymorphism of the major histocompatibility complex class I chain-related gene A; P1: Long COVID vs No long COVID; P2: Long COVID vs Control. Pc: P corrected by Bonferroni; n.s: not significant
