# Supplementary material for: Comprehensive Immunogenetic Analysis of Long COVID Reveals HLA‐B*15 as an Independent Protective Factor
Source: HLA. 2026 Nov 13;108(5):e71089. doi: 10.1111/tan.71089 (PMC13762371; doi:10.1111/tan.71089)
Supplement: Supplementary file 2 [file TAN-108-e71089-s002.docx]

**Supplementary Table 4.** Distribution of CCR5 Δ32 genotypes in patients with and without Long COVID.

|  | Long COVID (n=154) | No Long COVID (n=229) | p-value |
| --- | --- | --- | --- |
| WT/WT | 0.870 | 0.878 | n.s |
| WT/ Δ32 | 0.123 | 0.118 | n.s |
| Δ32/ Δ32 | 0.006 | 0.004 | n.s |

WT: wild type; Δ32: 32-base pair deletion; n.s: not significa
