# Supplementary material for: Comprehensive Immunogenetic Analysis of Long COVID Reveals HLA‐B*15 as an Independent Protective Factor
Source: HLA. 2026 Nov 13;108(5):e71089. doi: 10.1111/tan.71089 (PMC13762371; doi:10.1111/tan.71089)
Supplement: Supplementary file 3 [file TAN-108-e71089-s003.docx]

**Supplementary Table 2.** Distribution of Bw4/Bw6 epitopes and HLA-C C1/C2 groups in patients with and without Long COVID and healthy controls.

|  | Long COVID (n=154) | No Long COVID (n=229) | Controls (n=959) | P1 (Pc) | P2 (Pc) |
| --- | --- | --- | --- | --- | --- |
| Homozygous Bw4 | 0.188 | 0.210 | 0.189 | n.s | n.s |
| Homozygous Bw6 | 0.266 | 0.183 | 0.182 | n.s | n.s |
| Heterozygous Bw4/Bw6 | 0.545 | 0.607 | 0.629 | n.s | n.s |
| Homozygous C1 | 0.292 | 0.332 | 0.316 | n.s | n.s |
| Homozygous C2 | 0.156 | 0.162 | 0.208 | n.s | n.s |
| Heterozygous C1/C2 | 0.552 | 0.507 | 0.476 | n.s | n.s |

P1: Long COVID vs No long COVID; P2: Long COVID vs Control. Pc: P corrected by Bonferroni; n.s: not significant.
