# Supplementary material for: Comprehensive Immunogenetic Analysis of Long COVID Reveals HLA‐B*15 as an Independent Protective Factor
Source: HLA. 2026 Nov 13;108(5):e71089. doi: 10.1111/tan.71089 (PMC13762371; doi:10.1111/tan.71089)
Supplement: Supplementary file 4 [file TAN-108-e71089-s004.docx]

**Supplementary Table 1.** Symptom checklist used for the clinical assessment of Long COVID.

**Clinical manifestation**

1. **Respiratory symptoms**

Cough

Dyspnea

Chest pain

More than one respiratory symptom

2. **Gastrointestinal symptoms**

Diarrhea

Abdominal pain

Constipation

More than one gastrointestinal symptom

3. **Cardiovascular symptoms**

Tachycardia/palpitations

Orthostatic intolerance

Hypertension

More than one cardiovascular symptom

4. **Neurological symptoms**

Brain fog

Memory impairment

Headache

More than one neurological symptom

Other neurological symptoms (tremor, paresthesias)

5. **Musculoskeletal symptoms**

Myalgia

Arthralgia

More than one musculoskeletal symptom

6. **Taste and/or smell disorders**

Dysgeusia

Hyposmia

Both dysgeusia and hyposmia

7. **Others**

Anxiety/Depression

Sleep disorders (insomnia)

Dizziness/Syncope

Fatigue/Asthenia

Post-exertional malaise
